# Unusual SARS-CoV-2 intrahost diversity reveals lineage superinfection

**DOI:** 10.1099/mgen.0.000751

**Published:** 2022-03-17

**Authors:** Filipe Zimmer Dezordi, Paola Cristina Resende, Felipe Gomes Naveca, Valdinete Alves do Nascimento, Victor Costa de Souza, Anna Carolina Dias Paixão, Luciana Appolinario, Renata Serrano Lopes, Ana Carolina da Fonseca Mendonça, Alice Sampaio Barreto da Rocha, Taina Moreira Martins Venas, Elisa Cavalcante Pereira, Marcelo Henrique Santos Paiva, Cassia Docena, Matheus Filgueira Bezerra, Laís Ceschini Machado, Richard Steiner Salvato, Tatiana Schäffer Gregianini, Leticia Garay Martins, Felicidade Mota Pereira, Darcita Buerger Rovaris, Sandra Bianchini Fernandes, Rodrigo Ribeiro-Rodrigues, Thais Oliveira Costa, Joaquim Cesar Sousa, Fabio Miyajima, Edson Delatorre, Tiago Gräf, Gonzalo Bello, Marilda Mendonça Siqueira, Gabriel Luz Wallau

**Affiliations:** ^1^​ Departamento de Entomologia, Instituto Aggeu Magalhães (IAM), FIOCRUZ-Pernambuco, Recife, Pernambuco, Brazil; ^2^​ Núcleo de Bioinformática (NBI), Instituto Aggeu Magalhães (IAM), FIOCRUZ-Pernambuco, Recife, Pernambuco, Brazil; ^3^​ Laboratory of Respiratory Viruses and Measles (LVRS), Instituto Oswaldo Cruz, FIOCRUZ-Rio de Janeiro, Rio de Janeiro, Brazil; ^4^​ Laboratório de Ecologia de Doenças Transmissíveis na Amazônia (EDTA), Instituto Leônidas e Maria Deane, FIOCRUZ-Amazonas, Manaus, Amazonas, Brazil; ^5^​ Núcleo de Ciências da Vida, Universidade Federal de Pernambuco (UFPE), Centro Acadêmico do Agreste, Caruaru, Pernambuco, Brazil; ^6^​ Núcleo de Plataformas Tecnológicas (NPT), Instituto Aggeu Magalhães (IAM), FIOCRUZ-Pernambuco, Recife, Pernambuco, Brazil; ^7^​ Departamento de Microbiologia, Instituto Aggeu Magalhães (IAM), FIOCRUZ-Pernambuco, Recife, Pernambuco, Brazil; ^8^​ Laboratório Central de Saúde Pública, Centro Estadual de Vigilância em Saúde da Secretaria de Saúde do Estado do Rio Grande do Sul (LACEN/CEVS/SES-RS), Porto Alegre, Rio Grande do Sul, Brazil; ^9^​ Centro Estadual de Vigilância em Saúde da Secretaria de Saúde do Estado do Rio Grande do Sul, Porto Alegre, Rio Grande do Sul, Brazil; ^10^​ Laboratório Central de Saúde Pública do Estado da Bahia (LACEN-BA), Salvador, Bahia, Brazil; ^11^​ Laboratório Central de Saúde Pública do Estado de Santa Catarina (LACEN-SC), Florianópolis, Santa Catarina, Brazil; ^12^​ Laboratório Central de Saúde Pública do Estado do Espírito Santo (LACEN-ES), Vitória, Espírito Santo, Brazil; ^13^​ Analytical Competence Molecular Epidemiology Laboratory (ACME), FIOCRUZ-Ceará, Fortaleza, Ceará, Brazil; ^14^​ Departamento de Biologia. Centro de Ciências Exatas, Naturais e da Saúde, Universidade Federal do Espírito Santo, Alegre, Espírito Santo, Brazil; ^15^​ Instituto Gonçalo Moniz, FIOCRUZ-Bahia, Salvador, Bahia, Brazil; ^16^​ Laboratório de AIDS e Imunologia Molecular, Instituto Oswaldo Cruz, FIOCRUZ-Rio de Janeiro, Rio de Janeiro, Brazil

**Keywords:** codetection, coinfection, COVID-19, genomics

## Abstract

Severe Acute Respiratory Syndrome Coronavirus 2 (SARS-CoV-2) has infected almost 200 million people worldwide by July 2021 and the pandemic has been characterized by infection waves of viral lineages showing distinct fitness profiles. The simultaneous infection of a single individual by two distinct SARS-CoV-2 lineages may impact COVID-19 disease progression and provides a window of opportunity for viral recombination and the emergence of new lineages with differential phenotype. Several hundred SARS-CoV-2 lineages are currently well phylogenetically defined, but two main factors have precluded major coinfection/codetection and recombination analysis thus far: (i) the low diversity of SARS-CoV-2 lineages during the first year of the pandemic, which limited the identification of lineage defining mutations necessary to distinguish coinfecting/recombining viral lineages; and the (ii) limited availability of raw sequencing data where abundance and distribution of intrasample/intrahost variability can be accessed. Here, we assembled a large sequencing dataset from Brazilian samples covering a period of 18 May 2020 to 30 April 2021 and probed it for unexpected patterns of high intrasample/intrahost variability. This approach enabled us to detect nine cases of SARS-CoV-2 coinfection with well characterized lineage-defining mutations, representing 0.61 % of all samples investigated. In addition, we matched these SARS-CoV-2 coinfections with spatio-temporal epidemiological data confirming its plausibility with the cocirculating lineages at the timeframe investigated. Our data suggests that coinfection with distinct SARS-CoV-2 lineages is a rare phenomenon, although it is certainly a lower bound estimate considering the difficulty to detect coinfections with very similar SARS-CoV-2 lineages and the low number of samples sequenced from the total number of infections.

## Impact Statement

Severe Acute Respiratory Syndrome Coronavirus 2 (SARS-CoV-2) is the etiological agent of the global pandemic that in approximately 2 years has caused a large public health emergency leading to the death of more than 5 million people worldwide. Despite the vast literature about the SARS-CoV-2 genomics, there is still a knowledge gap regarding the intrahost nucleotide diversity during SARS-CoV-2 infection and detection of coinfection and recombination of different viral lineages. Our results, based on the largest dataset of raw sequenced reads assembled so far from Brazil, shows nine coinfection events from patients of different Brazilian regions. Knowledge of these events allows a more detailed understanding of how we can identify them, its impact on disease progression, the likelihood of new recombining lineage emergence and early detection of circulating lineages before official reports.

## Data Summary

The raw fastq data of codetection cases are deposited on gisaid.org and are associated to the following GISAID codes: EPI_ISL_1068258, EPI_ISL_2491769, EPI_ISL_2491781, EPI_ISL_2645599, EPI_ISL_2661789, EPI_ISL_2661931, EPI_ISL_2677092, EPI_ISL_2777552, EPI_ISL_3869215. Supplementary Material are available on Figshare at https://doi.org/10.6084/m9.figshare.19361270.v1 [1]. The workflow code used in this study is publicly available on: https://github.com/dezordi/ViralFlow.

## Introduction

SARS-CoV-2, the etiological agent of the COVID-19 pandemic, has a relatively low mutation rate compared to other RNA viruses [2], and most viral lineages are normally defined by only a few synapomorphic SNPs (*n*<10) [3]. However, the pervasiveness of SARS-CoV-2 infections during the COVID-19 pandemic provided substantial opportunities for the virus to explore the fitness landscape through single nucleotide substitutions and/or indels, giving rise to a range of more transmissible variants of concern (VOCs). These lineages are characterized by an unusual pattern of lineage-defining SNPs along the genome (*n*>15) [4–6].

Coinfection is defined as a single host infection by more than one pathogen or virus lineage simultaneously. Despite being a rare phenomenon, it may provide opportunity for genetic recombination, an event known to occur in viruses of the *Coronaviridae* family [7, 8] including SARS-CoV-2-like viruses [9]. Recombinant viruses may, in turn, trigger the emergence of new lineages with enhanced biological properties, including the capacity to infect new hosts (expansion of viral host range) [10–13]. The frequency of coinfection and its role to promote recombination-driven SARS-CoV-2 evolution and the emergence of SARS-CoV-2 lineages is still poorly understood. The low variability found in SARS-CoV-2 lineages and the few well-defined lineage-specific SNPs until the second half of 2020 probably hindered the identification of coinfection and recombination events of SARS-CoV-2 lineages so far. In contrast, the emergence of VOC lineages carrying a substantial number of additional SNPs may provide enough markers to currently detect these events. A number of coinfection cases were reported for SARS-CoV-2, including lineages B.1.1.28/B.1.1.33 and B.1.1.91/B.1.1.28 [14] in Brazil, several variants of interest (VOIs) and VOCs [15], and different lineages in the UK [16, 17]. Moreover, putative coinfections were indirectly inferred from North America and Europe patients by detecting recombinant genomes [18–20].

In this study, we assessed amplicon sequencing reads of 2263 SARS-CoV-2 samples from Brazilian patients generated by the Fiocruz Genomic Surveillance Network. We identified nine coinfection cases through the identification of an unusual pattern of intrahost single nucleotide variant (iSNV) sites and phylogenetic reconstruction of alternative SARS-CoV-2 genomes generated from well supported major and minor allele frequency nucleotide variants. Moreover, epidemiological trends of circulating lineages in each Brazilian state supported that the SARS-CoV-2 VOIs and VOC lineages found in these coinfected samples were also cocirculating at the time of sampling, thus providing further plausibility for our findings.

## Methods

### SARS-CoV-2 sequences and ethical aspects

The sequencing data was obtained from the genomic survey of SARS-CoV-2 positives samples sequenced by FIOCRUZ’s Genomic Surveillance Network between 18 May 2020 and 30 April 2021. SARS-CoV-2 genomes were amplified and sequenced using previously described Illumina protocols [21–23] (Table S1, available in the online version of this article). The frequency of lineages obtained from Brazilian states was evaluated using data recovered from GISAID (gisaird.org) on 23 July 2021.

### Genome assembly and intrahost variant analysis

The genomic analysis were performed with ViralFlow v.0.0.5 [24], through the following steps: removal of duplicated reads, adapters and read extremities with less than 20 of phred score quality with the fastp tool [25]; reference genome assembly using BWA [26] to map reads against the SARS-CoV-2 Wuhan reference genome (NC_045512.2); the consensus genomes generation with samtools mpileup [27] and iVar [28], using a threshold quality score of 30 and calling SNPs and indels present as major allele frequencies. After the consensus generation, the bam-readcount tool [29] retrieve the proportion of each base (A, C, T, G) present in each position of the bam file and an *in house* python script (intrahost.py) identifies iSNV sites following the specific rules: the minor variant (MinV) should represent at least 5 % of total position depth and should appear in both sense and antisense reads (at least 5 % in each sense) with a depth of at least 100 reads. Two consensus genomes were generated as output based on the major and minor allele frequency of each iSNV site: the major variant (MajV) with the nucleotide present in major allele frequency in each genomic position, and the MinV with the nucleotide present in the lower allele frequency at that same positions. The consensus genomes were automatically analysed with PangoLineage v1.1.23 with pangoLEARN updated at 28 May 2021 [30] and to Nextclade [31] tools. Only genomes with more than 95 % coverage breadth and 100 reads of average coverage depth (Table S2) were considered.

If the MajV and MinV genomes were assigned to the same viral lineage, we may assume that the variability observed likely resulted from: (I) *de novo* generation of intrahost variants that emerged during viral replication; or (II) coinfection with two viruses of the same lineage. Conversely, if MajV and MinV genomes were assigned to different lineages, the intrahost variability observed is more likely derived from a codetection event. All samples in which alternative genomes where assigned to different pango lineages were manually curated with Interactive Genomic Viewer [32], indels related to intrahost variants into specific genomes that change the coding frame were discarded. Additional evidence of codetection was searched on the raw sequence reads: (I) if the proportion of reads supporting lineage-specific defining SNPs are similar it suggests codetection while if the proportion is drastically different the variability is likely derived from *de novo* intrahost variability; (II) if SNPs and/or intrahost variants are restricted to some specific SARS-CoV-2 genomic region it likely indicates a recombination event. Otherwise, if iSNV sites are distributed along the entire SARS-CoV-2 genome it is likely to be derived from the codetection of different SARS-CoV-2 genomes in the same sample.

### Recombination analysis

To identify putative recombination events, we compared the set of mutations present in each genome with the expected set of mutations of the lineage assigned by PANGO lineage. In this step, the common mutations (present in at least 75 % of genomes per lineage deposited on GISAID) in the 33 lineages identified in our samples are established based in the Lineage|Mutation Tracker available on outbreak.info, updated on 08 November 2021. The excess or lack of mutations are then compared with the mutations annotated with NextClade using an *in house* R script (https://github.com/dezordi/SARS-CoV-2_tools/ blob/master/compare_mutation.R). Samples with qc.overallStatus equal to ‘good’ and with ten amino acid mutations missing or in excess, were separated to a manual curation of lineage-specific mutations with the same information from outbreak.info. Samples with signals of mutations from two different lineages (parental lineages) were then analysed for recombination following the methodology of previous published studies [18, 19].

### Phylogenetic analysis

A reference alignment was created using mafft [33] with the 6167 genomes, which represents the genomes used in Nextstrain [34] global phylogeny with N content less than 5 % accessed on 24 May 2021 and Brazilian genomes obtained through a cd-hit-est [35] clusterization of genomes present on GISAID at 16 March 2021 with high-quality and with more than 99.8 % sequence identity and from the same Brazilian state. The reference alignment was edited to mask UTR regions and to maintain the indel regions. The 32 MajV and MinV consensus genomes were aligned to reference alignment with mafft add, and we performed a maximum-likelihood phylogenetic analysis with IQtree2 [36] employing the aLRT branch support evaluation method and the GTR+F+R5 nucleotide substitution model. The PANGO lineages were evaluated with pangolin and used to annotate the tree with iTOL [37].

## Results and discussion

Our initial analysis revealed that 1462 out of 2263 genomes had enough sequencing breadth and depth to be able to consistently detect and characterize the viral genomic variability at the sequencing reads level. In total, 1150 out of 1462 SARS-CoV-2 positive samples investigated showed at least one iSNV site, that is, at least one genomic position with more than 100 reads supporting a minimum of two alternative nucleotides. Those samples showed an average coverage depth of 1817.46 (sd=908.59) and an average coverage breath supported by at least 100 reads of 99.66 (sd=1.10) (Table S2). In addition, we estimated a mean of 2.57 iSNVs per genome (Table S3). MajV and MinV consensus sequences representing the viral genome variability found in each sample were generated for all samples bearing well supported alternative nucleotides and then assessed for lineage assignment using the PANGOlineage tool.

**Fig. 1.**
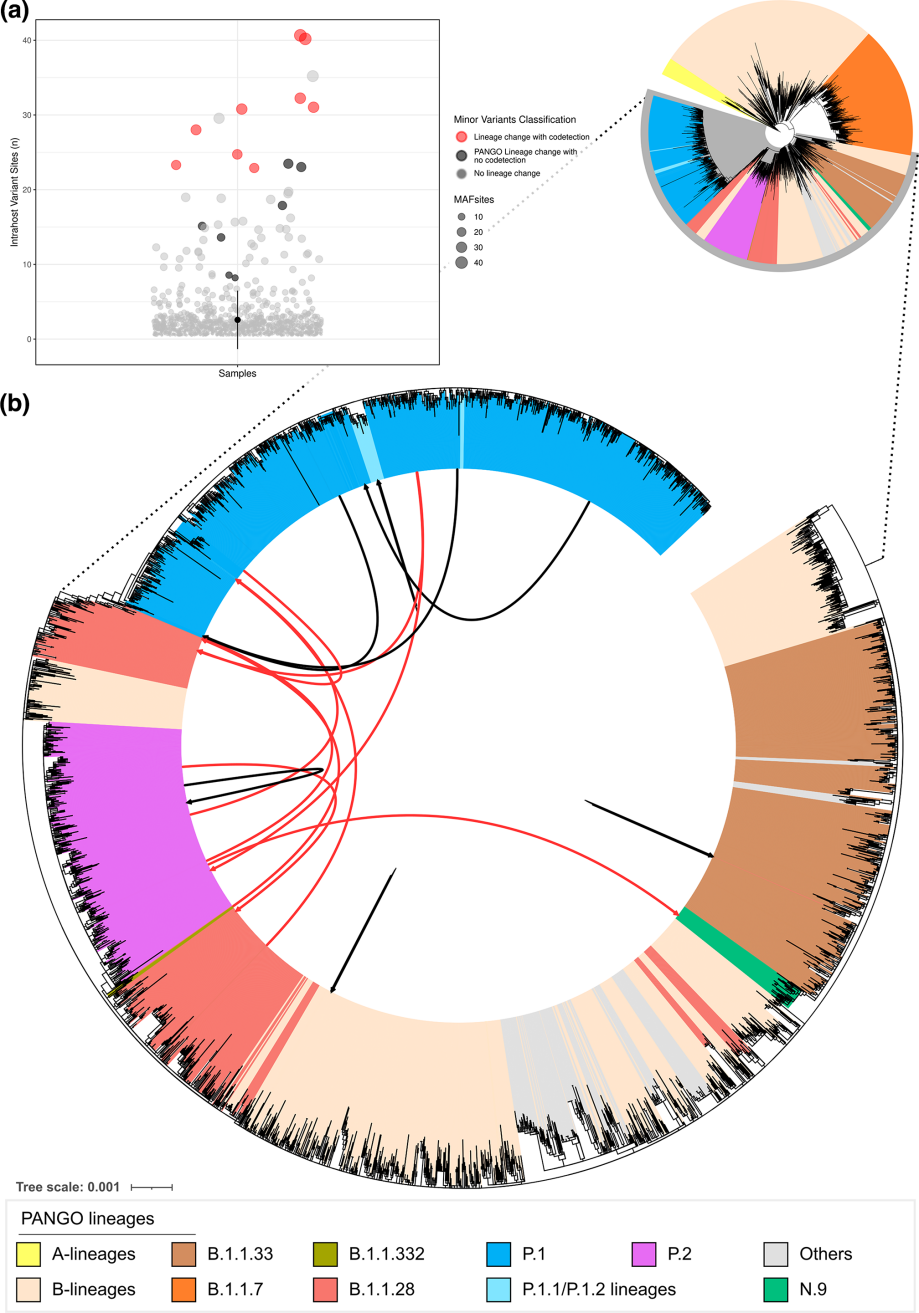
Number of intrahost variant sites from 1150 SARS-CoV-2 samples and phylogenetic analysis of alternative MajV and MinV consensus genomes recovered from the same sample. (a) Dot plot with number of iSNVs per sample; (b) maximum-likelihood phylogenetic tree. Others: R, S, U, L, D, C lineages. Red arrows represent samples with alternative genomes showing lineage change while black arrows indicate alternative samples with no lineage change.

We detected 16 instances in which MajV and MinV were assigned to distinct lineages (iSNV sites: mean=24, sd=9.75), including former VOIs and now known as variants for further monitoring (VFM) N.9 and P.2 as well as the VOC P.1 (Table S4). To further confirm the lineage assigned by the PANGOlineage tool, we performed a phylogenetic analysis of representative lineages including both MajV and MinV genomes. Nine alternative genomes were confidently repositioned into distinct lineages (Fig. 1, red arrows, mean iSNVs sites 30.44, sd=6.63), while the remaining alternative genomes branched within the same lineage (Fig. 1, black arrows, mean iSNVs 23.06, sd=10.54). Seven out of nine putative coinfection events involve the VOC Gamma (P.1 lineage) (Table 1). In four cases, Gamma (lineage P.1) represented the MajV genome, while in the remaining three cases, it corresponded to the MinV genome. The large proportion of codetection events with P.1/Gamma is likely a result of the higher number of lineage-defining SNPs characteristic of this VOC that facilitate the distinction between coinfecting SARS-CoV-2 lineages. As more distinct lineages, bearing many lineage-defining SNPs, coinfect the same host, it becomes increasingly more likely to objectively distinguish coinfections through the reconstruction of alternative intrasample viral genomes.

**Table 1.** Summary of coinfection events

Sample	State	City	iSNVs	Breadth*	Depth†	Lineages‡	Collection date	First MajV§ lineage available on GISAID	First MinV§ lineage available on GISAID
CE-FIOCRUZ-00657	CE	Fortaleza	23	98.95	762.04	P.2/P.1	2021-01-20	2020-11-20/2020-04-15	2021-01-07/2021-01-07
AM-FIOCRUZ-21142481RG	AM	Manaus	31	98.9	1105.46	P.1/B.1.1.28	2021-01-13	2020-12-03/2020-12-03	2020-04-13/2020-04-13
RS-FIOCRUZ-2060	RS	Canoas	25	99.69	3509.92	P.2/B.1.1.28	2021-01-07	2021-01-26/2020-08-31	2020-05-21/2020-03-16
BA-FIOCRUZ-4739	BA	Salvador	31	99.73	5354.25	P.2/N.9	2021-01-08	2020-10-26/2020-06-26	2020-12-10/2020-11-12
ES-FIOCRUZ-6993	ES	Aracruz	32	99.67	2222.7	B.1.1.28/P.1	2021-01-09	2020-10-13/2020-10-13	2021-04-09/2021-01-22
CE-FIOCRUZ-6559	CE	Fortaleza	41	99.85	4090.76	P.1/P.2	2021-01-24	2021-01-07/2021-01-07	2020-11-20/2020-04-15
SC-FIOCRUZ-10891	SC	Porto Belo	28	98.72	2153.25	B.1.1.332/B.1.1.28	2021-02-22	2021-02-22/2021-02-22	2020-03-18/2020-03-18
BA-FIOCRUZ-10781	BA	Salvador	40	99.73	1996.63	P.2/P.1	2021-02-10	2020-10-26/2020-06-26	2020-12-27/2020-12-27
AM-FIOCRUZ-21890619RGS	AM	Manaus	23	96.59	1018.89	P.1/B.1.1.28	2021-01-13	2020-12-03/2020-12-03	2020-04-13//2020-04-13

*Coverage breadth supported by 100 reads.

†Average coverage depth. AM: Amazonas; BA: Bahia; ES: Espírito Santo; CE: Ceará; RS: Rio Grande do Sul; SC: Santa Catarina.

‡MajV/MinV Pango lineages supported by the the phylogenetic analysis.

§Date of the first genome deposited on GISAID of each variant into the specific municipality/state, updated on 26 July 2021.

**Fig. 2.**
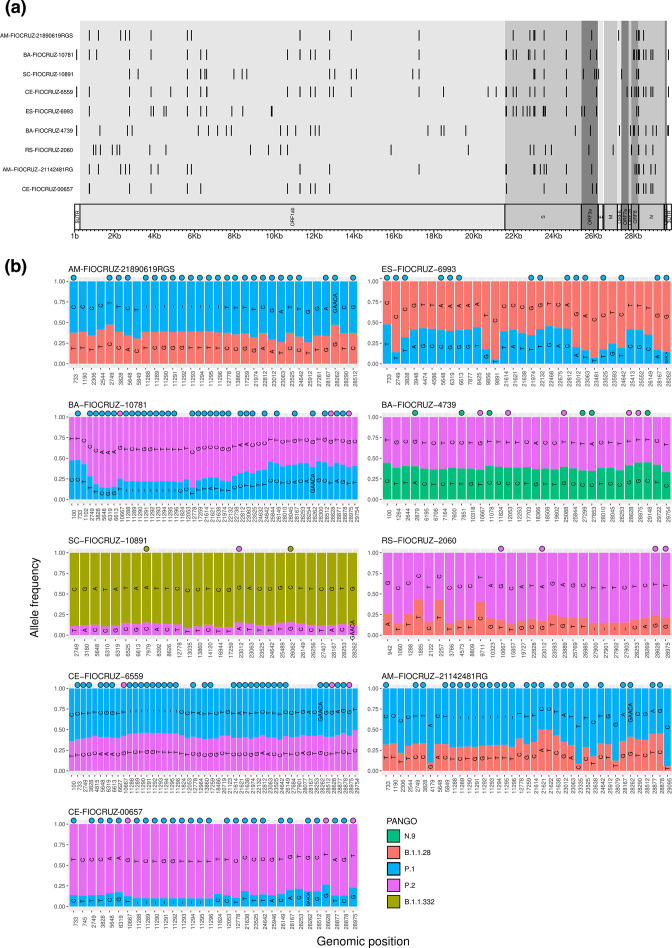
MajV and MinV of samples with codetection of different SARS-CoV-2 lineages. (a) Karyoploter with iSNV sites across the SARS-CoV-2 genome. (b) iSNV sites with read-depth frequency supporting MajV and MinV. Defining SNPs based on data of outbreak.info update on 24 July 2021, are indicated with a circle. Karyoplots depicting iSNV-site sequencing depth can be accessed in File S1, and raw depth values can be accessed in Table S5.

Intrahost single nucleotide variant sites identified showed several lineage defining SNPs spread across the whole SARS-CoV-2 genome, and the sequencing read depth was roughly similar throughout the genome (Fig. 2a, Table S5). Considering the lineage defining SNPs present in lineages assigned into MajV and MinV genomes and the absence of lineage defining SNPs of different lineages interlaced in the same genome (Fig. 2b, Table S3), our results indicate that the coinfection cases detected here did not generate hybrid recombinant genomes. Moreover, the analysis of missing and extra mutations of all 1150 consensus genomes did not reveal any putative recombined genome (Table S6). In order to assess if codetection could be a result of sample contamination we reassessed sample AM-FIOCRUZ-21142481RG from RNA extraction, library preparation and sequencing. We confirmed the intrahost variability for 25 out of 31 sites present in the first sequencing run (Table S7). Lineage assignment, phylogenetic reconstruction and the detection of SNP defining mutations confirmed the codetection status of that sample (Table S4) suggesting that the intrahost variability found in this sample did not result from laboratory contamination. However, this reassessment did not rule out contamination during sample collection and cannot be extrapolated to the other eight coinfection samples.

**Fig. 3.**
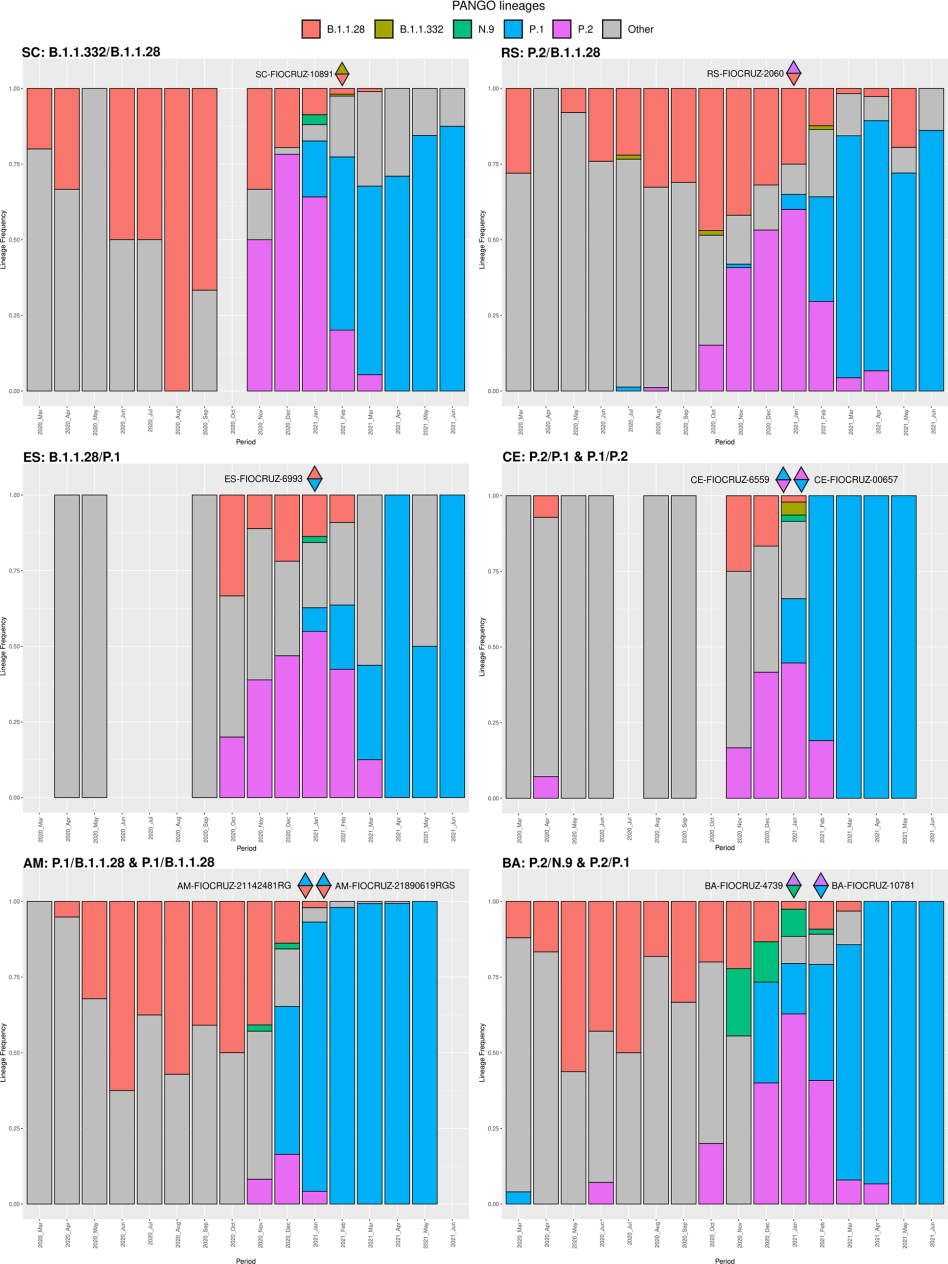
SARS-CoV-2 lineage proportion through time in different Brazilian states with codetection cases. Data were recovered from GISAID on 23 July 2021, raw data can be accessed in Table S8. Upper triangles coloured with the lineage of major consensus genomes and lower triangles with minor consensus genomes lineages.

The plausibility of the codetection events was further validated by the fact of all MajV and MinV alternative genome lineages identified by our study were cocirculating in their sampling location, overlapping in time and space, as well as matching with SARS-CoV-2 lineage information form their respective geographical states (Fig. 3). The MajV genome corresponding to the predominant lineages circulating in Rio Grande do Sul, Bahia and Amazonas states were recovered in our analysis. On the other hand, in Santa Catarina, both lineages involved in codetection were present at lower frequency than the dominant lineages at the same location and period of sampling. Only one event of VOC circulation without previous notification was detected, the VOC Gamma was detected as a MinV genome in sample ES-FIOCRUZ-6393 from Aracruz city, Espirito Santo state, collected on 9 January 2021. The earliest record on GISAID of the VOC Gamma in Aracruz municipality was on 9 April 2021 and in Espirito Santo was on 22 January 2021 (Table 1). Of note, the genomic sequence of this specimen available on GISAID under EPI_ISL_2645599 code confirmed the assignment of the MajV genome to lineage B.1.1.28. These findings highlight that the analysis of MinV genome revealed the early spread of cryptically circulating lineages not detected by the analysis of MajV consensus genomes alone.

Finally, the antiviral mechanism mediated by APOBEC-like host proteins against SARS-CoV-2 is known to induce a high frequency of ‘C→U transition’ in SARS-CoV-2 genomes [38] and may affect the identification of coinfection events based on the analysis of SARS-CoV-2 intrahost diversity. Our results showed a twofold difference of ‘C→U’ with respect to U→C or G→U mutations, a fourfold difference with respect to G→A or A→G and a 16-fold difference when compared with other transitions and transversions in single infection samples (File S2a), which is in line with other findings [16, 38]. By contrast, the frequency of intrahost changes in the nine coinfection cases showed a similar proportion between C→U and U→C (File S2b) mutations, suggesting that several lineage defining SNPs correspond to non-C→U mutations. Therefore, although the C→U bias has likely blurred the distinction of some lineage defining mutations, the remaining non-C→U lineage defining mutations are still sufficient to detect such events [38].

## Conclusions

In line with other studies, we showed that SARS-CoV-2 has a low intrahost variability overall. Our in-depth analysis revealed at least nine codetection events, which are corroborated by epidemiological data from cocirculating lineages in different Brazilian states. Codetection/coinfection events occurred at a lower rate in Brazil (0.61%) compared to Europe (1–4 %) [16, 17]. However, this is certainly a lower bound estimate due to the limitation of detecting coinfection events with the same viral lineage or with low-divergent viral lineages that dominated the first year of the pandemic. The large number of genomic sites carrying alternative nucleotides in codetection events may generate artificial hybrid consensus genomes, therefore a careful inspection of consensus sequences against sequencing read polymorphisms is warranted to generate robust consensus sequences. Considering the large case numbers of SARS-CoV-2 infections worldwide and that coinfection are more likely to happen in high-transmission settings, all efforts should be placed to limit SARS-CoV-2 transmission hence reducing the likelihood of coinfection and the probability of emergence of novel recombinant hybrid lineages with altered phenotype.

## Supplementary Data

Supplementary material 1

Supplementary material 2

Supplementary material 3

Supplementary material 4
