# Supplementary material for: Unusual SARS-CoV-2 intrahost diversity reveals lineage superinfection
Source: Microb Genom. 2022 Mar 17;8(3):000751. doi: 10.1099/mgen.0.000751 (PMC9176291; doi:10.1099/mgen.0.000751)
Supplement: Supplementary material 1 [file mgen-8-0751-s001.pdf]

CE-FIOCRUZ-00657

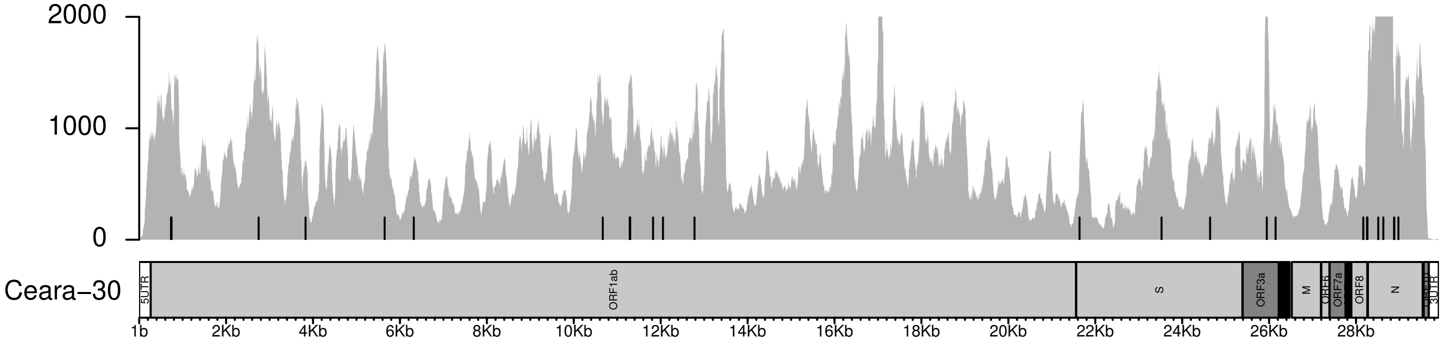

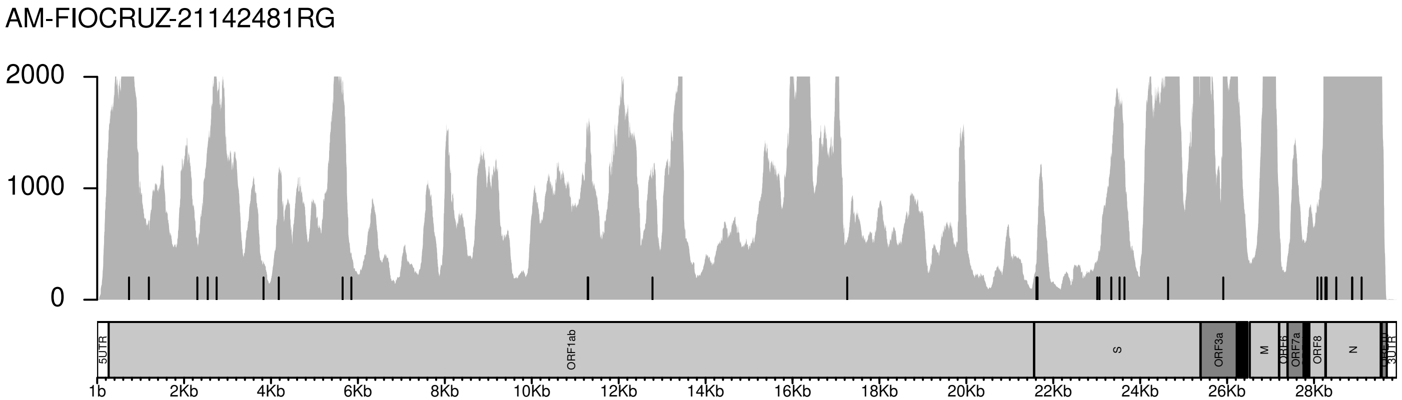

AM-FIOCRUZ-21142481RG
2000
1000
0
5UTR
ORF1ab
S
ORF3a
M
ORF6
ORF7a
ORF8
N
3UTR
1b
2Kb
4Kb
6Kb
8Kb
10Kb
12Kb
14Kb
16Kb
18Kb
20Kb
22Kb
24Kb
26Kb
28Kb

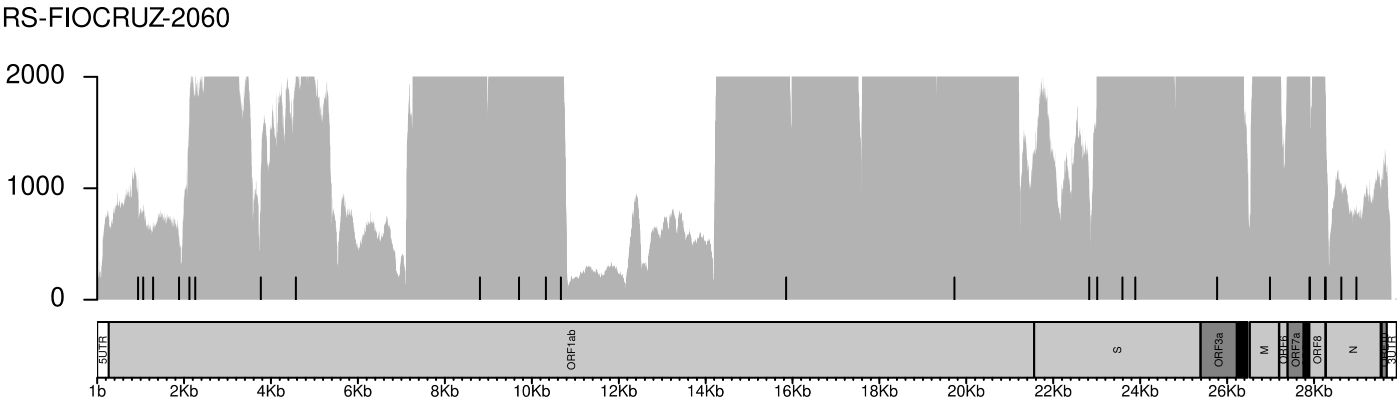
RS-FIOCRUZ-2060
2000
1000
0
5UTR
ORF1ab
S
ORF3a
M
ORF6
ORF7a
ORF8
N
3UTR
1b
2Kb
4Kb
6Kb
8Kb
10Kb
12Kb
14Kb
16Kb
18Kb
20Kb
22Kb
24Kb
26Kb
28Kb

BA-FIOCRUZ-4739

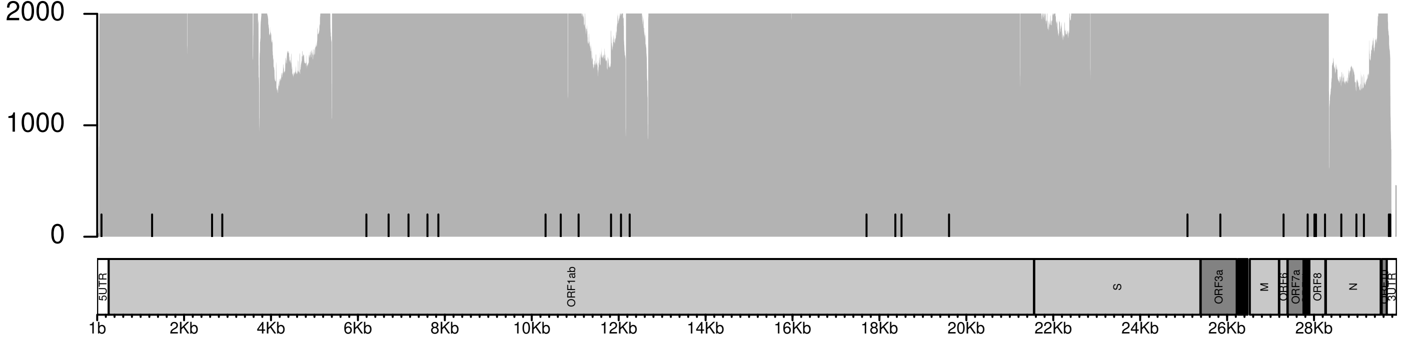

ES-FIOCRUZ-6993

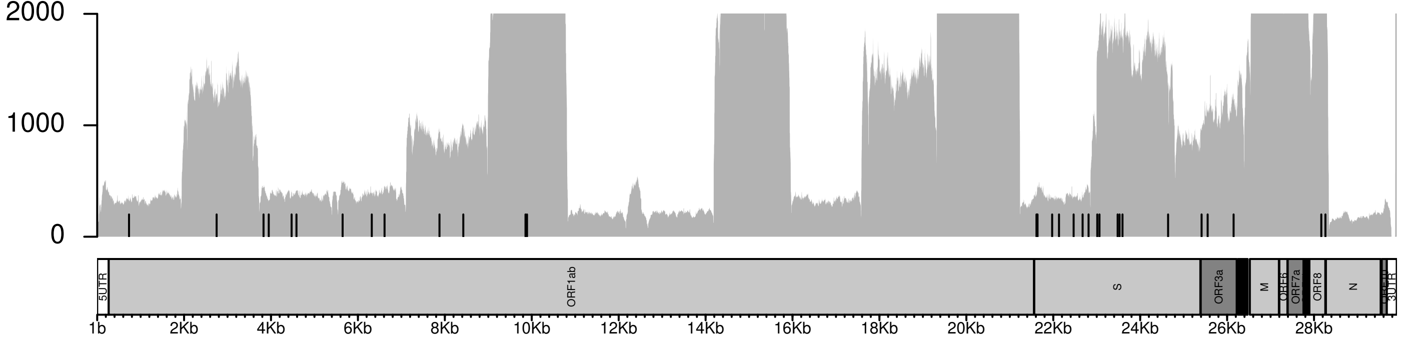

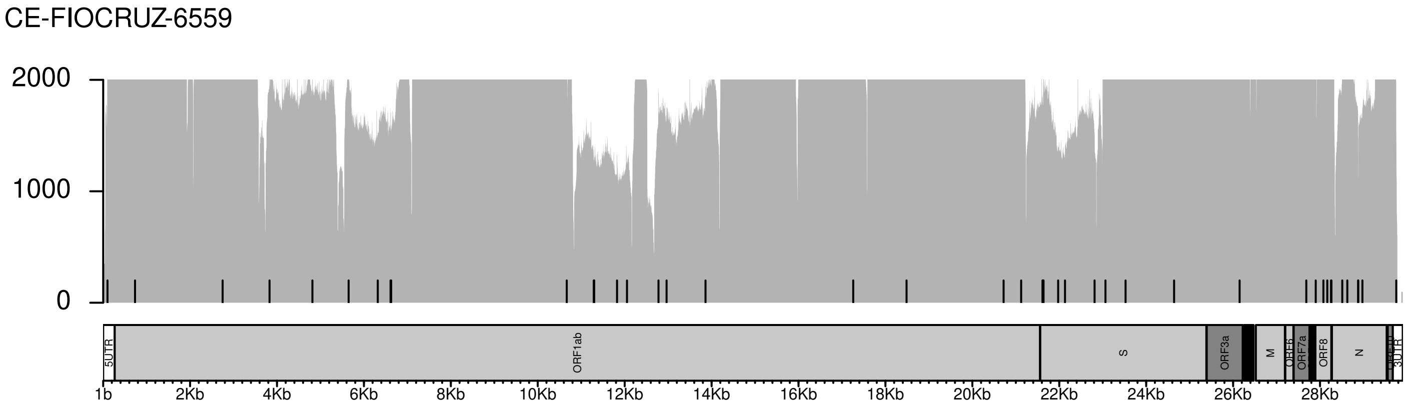

CE-FIOCRUZ-6559
2000
1000
0
5UTR
ORF1ab
S
ORF3a
M
ORF6
ORF7a
ORF8
N
3UTR
1b
2Kb
4Kb
6Kb
8Kb
10Kb
12Kb
14Kb
16Kb
18Kb
20Kb
22Kb
24Kb
26Kb
28Kb

SC-FIOCRUZ-10891

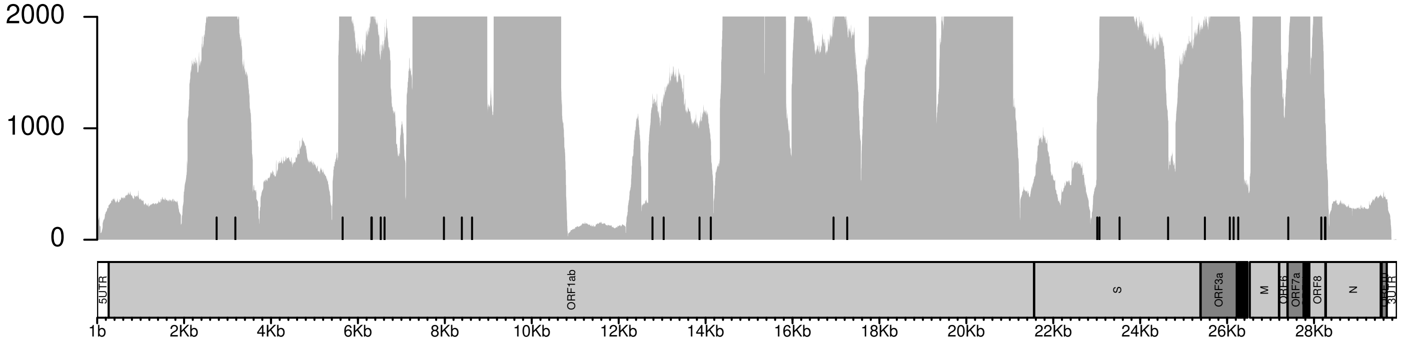

BA-FIOCRUZ-10781

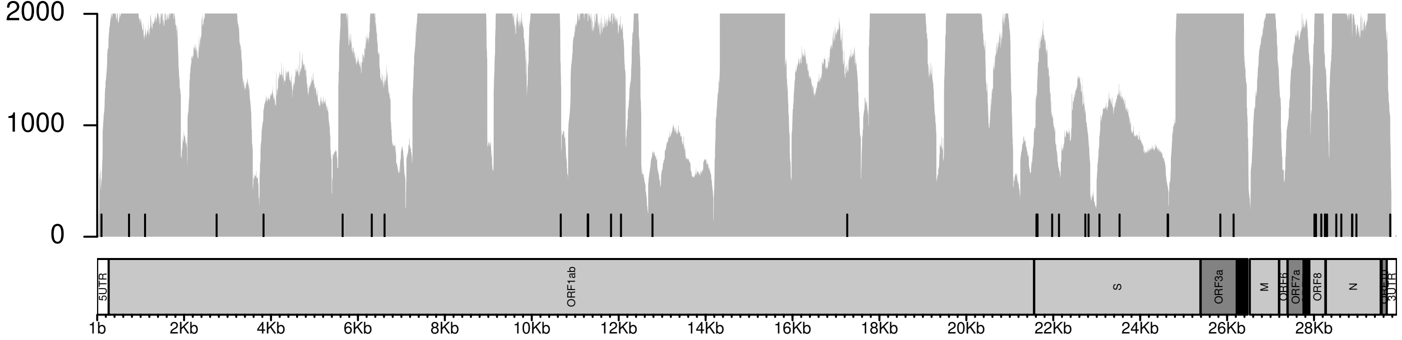

AM-FIOCRUZ-21890619RGS

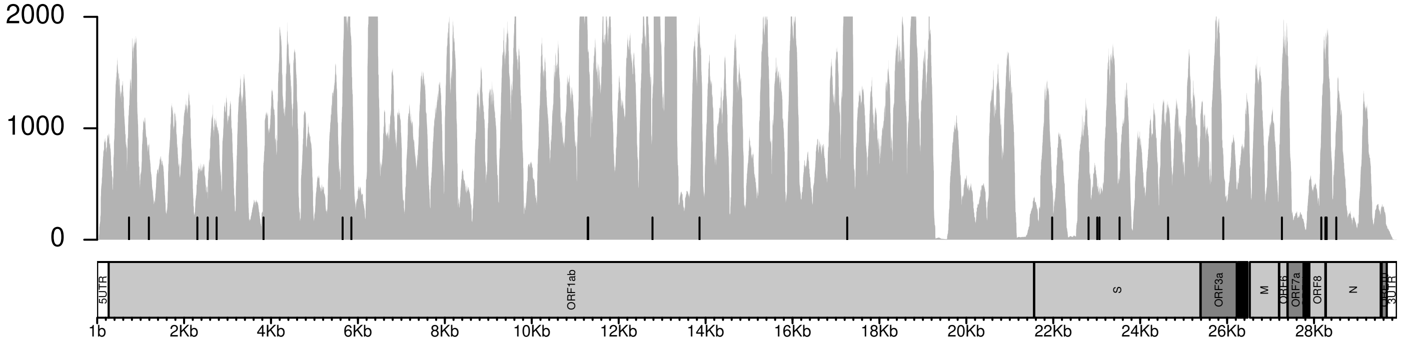

Supplementary File 1 - Black vertical bars represents minor allele frequency sites. Y axis represents read depth.
