# Supplementary material for: Unusual SARS-CoV-2 intrahost diversity reveals lineage superinfection
Source: Microb Genom. 2022 Mar 17;8(3):000751. doi: 10.1099/mgen.0.000751 (PMC9176291; doi:10.1099/mgen.0.000751)
Supplement: Supplementary material 2 [file mgen-8-0751-s002.pdf]

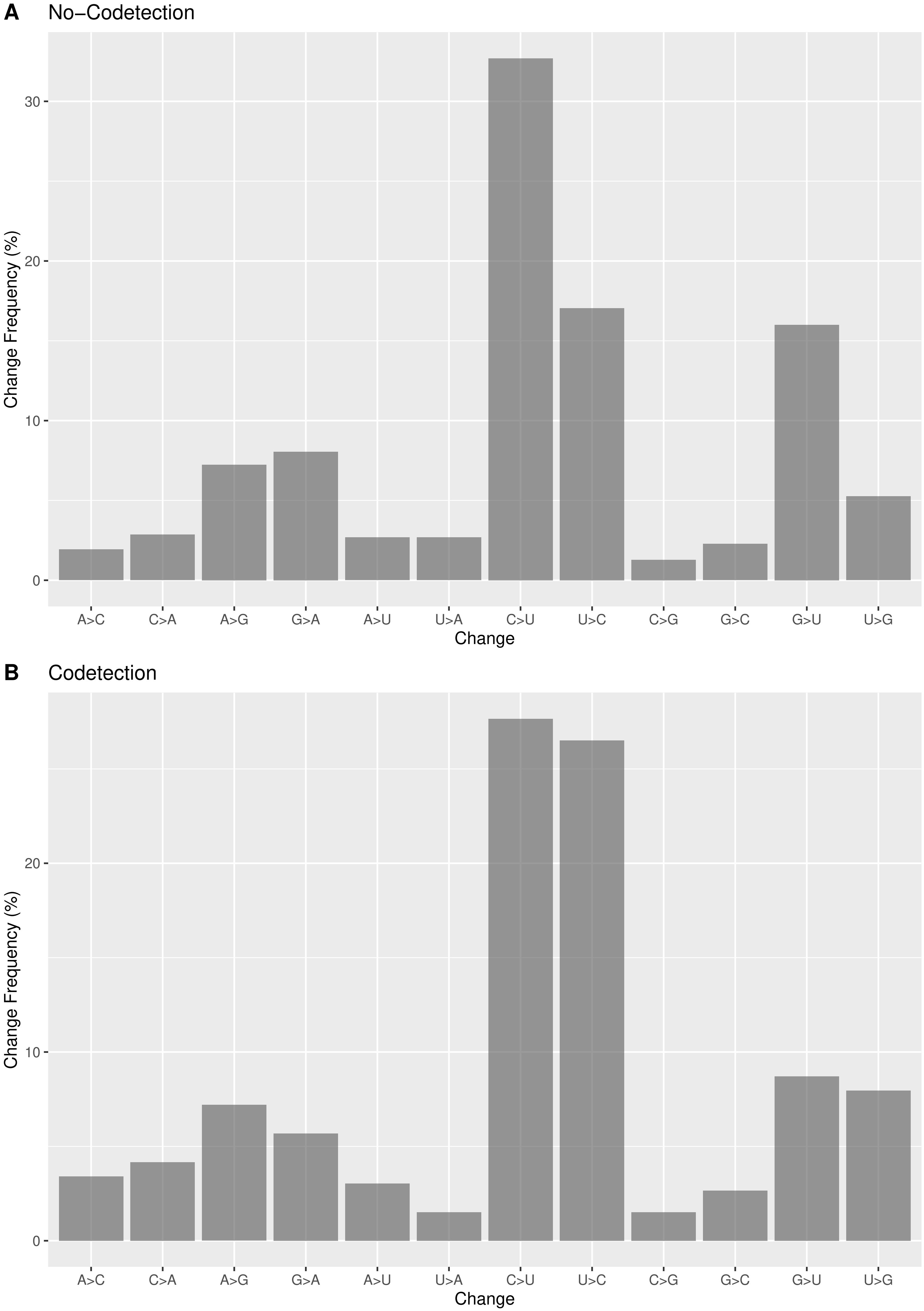

Supplementary File 2 - Distribuition of nucleotide changes between MajV and MinV genomes. **A.** Samples without co-detection signal. **B** Samples with co-detection signal.
